# Precise detection of circular dichroism in a cluster of nano-helices by photoacoustic measurements

**DOI:** 10.1038/s41598-017-05193-4

**Published:** 2017-07-12

**Authors:** Alessio Benedetti, Badrul Alam, Marco Esposito, Vittorianna Tasco, Grigore Leahu, Alessandro Belardini, Roberto Li Voti, Adriana Passaseo, Concita Sibilia

**Affiliations:** 1 S.B.A.I. Department, Physics Section, “Sapienza: Università di Roma”, Via A. Scarpa14/16, I-00161 Rome, Italy; 2 D.I.E.T. Department, “Sapienza: Università di Roma”, Via Eudossiana 18, I-00184 Rome, Italy; 3 CNR Nanotec, Istituto di Nanotecnologia, Polo di Nanotecnologia, c/o Campus Ecotekne, Via Monteroni, I-73100 Lecce, Italy; 4 “Università del Salento”, Dip. Mat-Fis “Ennio De Giorgi”, Via Arnesano, I-73100 Lecce, Italy

## Abstract

Compact samples of nano-helices built by means of a focused ion beam technology with large bandwidth and high dichroism for circular polarization are promising for the construction of built-in-chip sensors, where the ideal transducer must be sufficiently confined without compromising its filtering ability. Direct all-optical measurements revealed the sample’s dichroic character with insufficient details because of scattering and diffraction interference. On the other hand, photoacoustic measurements resulted to be a possible alternative investigation, since they directly deal with absorbed power and allow to get clear evidences of the differential selection for the two opposite polarization states. Multi-level numerical simulations confirmed the experimental results, proving once again the reliability of photoacoustic technique and the versatility of this class of dichroic artificial materials.

## Introduction

In the last years, the ultimate nanofabrication frontier represented by 3D nanostructures is generating promising and versatile novel nano-photonics devices. In particular, 3D nanostructures with broken symmetry enable exciting complex interactions with chiral light, generating the typical forms of the optical activity, such as the optical rotation (OR), i.e., the polarization plane rotation of the incident light, and the circular dichroism (CD)^1, 2^, i.e., the different absorption levels for left and right circularly polarized (CP) waves^3^. Among the possible 3D chiral geometries, the helix architecture represents, because of its intrinsic chirality, an effective choice to manifest detectable chiral effects^4–8^. However, the engineering of the chiro-optical properties, such as the frequency as well as the absorbed and scattered portions of left- and right-handed circularly polarized light, requires the full control on the geometrical and spatial parameters and on material composition of the 3D nanostructures. Hence, great efforts have been made to develop flexible nanofabrication techniques for the realization of helical based structures with nanometer accuracy^9^. Recently, focused ion and electron beam induced deposition (FIBID/FEBID) have demonstrated the effective capability to tailor helical nanostructures as a function of application-driven chiro-optical properties^10–14^, leading to the nanometer scale controlled fabrication of helix-shaped metal and dielectric nanostructures, organized in dense and ordered arrays of few micron area. Moreover, the FIBID/FEBID approach is particularly suitable for the building and the spatial localization of compact nano-devices as parts of embedded systems, such as built-in-chip sensors and integrated optoelectronic filters, where it is of key importance the integration of nanostructured materials onto small areas.

When dealing with 3D nanostructured samples with limited patterned area, commonly used all optical (AO) measurements, though requiring low power levels of inspecting light, are heavily altered by the electromagnetic (e.m.) field escaping from the un-patterned substrate region, as well as by scattering effects. On the other hand, the photoacoustic (PA) technique, based on the generation of heat when a sample absorbs an incoming light beam^15, 16^, allows for the exclusive extraction of information from 3D nanostructures without being polluted by signals coming from the non-absorbing substrate. Sound- and heat-based approaches like PA^17–20^, acousto-optic^21^ and photothermal techniques already demonstrated to be successful for the detection of CD in other chiral media and for high precision imaging^22^. Here we extend this research to artificially structured materials, since PA technique already demonstrated to be reliable for the measurement of chiroptical effects in nanostructured samples^23^.

In this paper, the CD of limited area nanohelices array fabricated by FIBID is studied by exploiting PA techniques, in addition to conventional all-optical (AO) measurements. This sample has been engineered in order to preserve the circular dichroism related to the helices’ natural chirality and, at the same time, to limit the physical in-plane extension, the high precision measurements obtained by PA method allow to overcome physical limitations related to the natural diffraction of light, and combined with the large and tailorable chiro-optical effects demonstrated by this kind of artificial materials, concur to the development of novel photonic devices. By considering the precise internal structure of the helices’ filament, numerical simulations have been performed to calculate the permittivity spectrum of the helix constitutive material, then we calculated the absorption spectra of the helices array, whose high CD levels confirmed the experimental results.

## Sample description and experimental measurements

**Figure 1:**
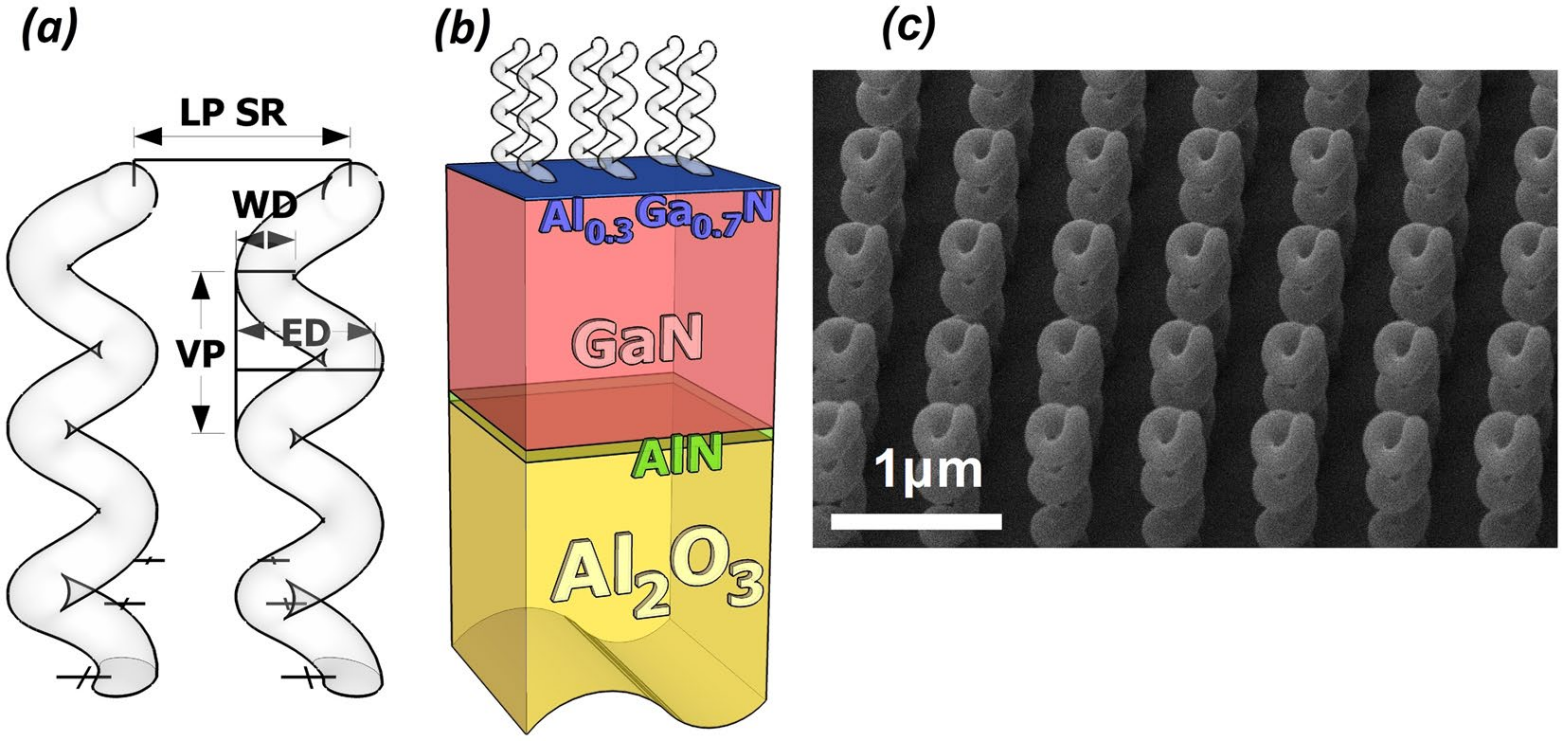
(**a**) Schematic view of the single helix, with another neighbor element. The wire diameter (WD) is 110 nm, the external diameter (ED) is 300 nm, and the vertical pitch (VP) is 300 nm. The helix is supposed to have a perfectly round shaped cross section. The lattice period (LP) of the square array is 700 nm. (**b**) Schematic view of the substrate laying beneath the helices layer. It consists of a GaN/AlGaN heterostructure where a two dimensional electron gas allows for charge effect management during the helices growth by FIBID^10, 24^. All the heterostructure is epitaxially grown on a thick (380 micron) Al2O3 substrate. (**c**) scanning electron microscope (SEM) view of the sample.

We investigated a sample consisting of an array of identical nano-helices designed to operate at near infrared (NIR) and visible (VIS) spectral regions, and realized by FIBID technique. Details on the helix construction can be found in refs 10 and 14. The helices consist of platinum-gallium-carbon (Pt-Ga-C) mixture, and are progressively grown upon a transparent dielectric multilayered substrate with a conductive layer^10^. The helices are localized at the nodes of a square-based array with a 700 nm lattice period (LP), covering a square area of 40 × 40 units (approximately with a side length of 28 µm). The single helix has three loops (N = 3), a wire section diameter (WD) of 110 nm, an external diameter (ED) of 300 nm, and the vertical pitch (VP) of 300 nm (Fig. 1).

**Figure 2:**
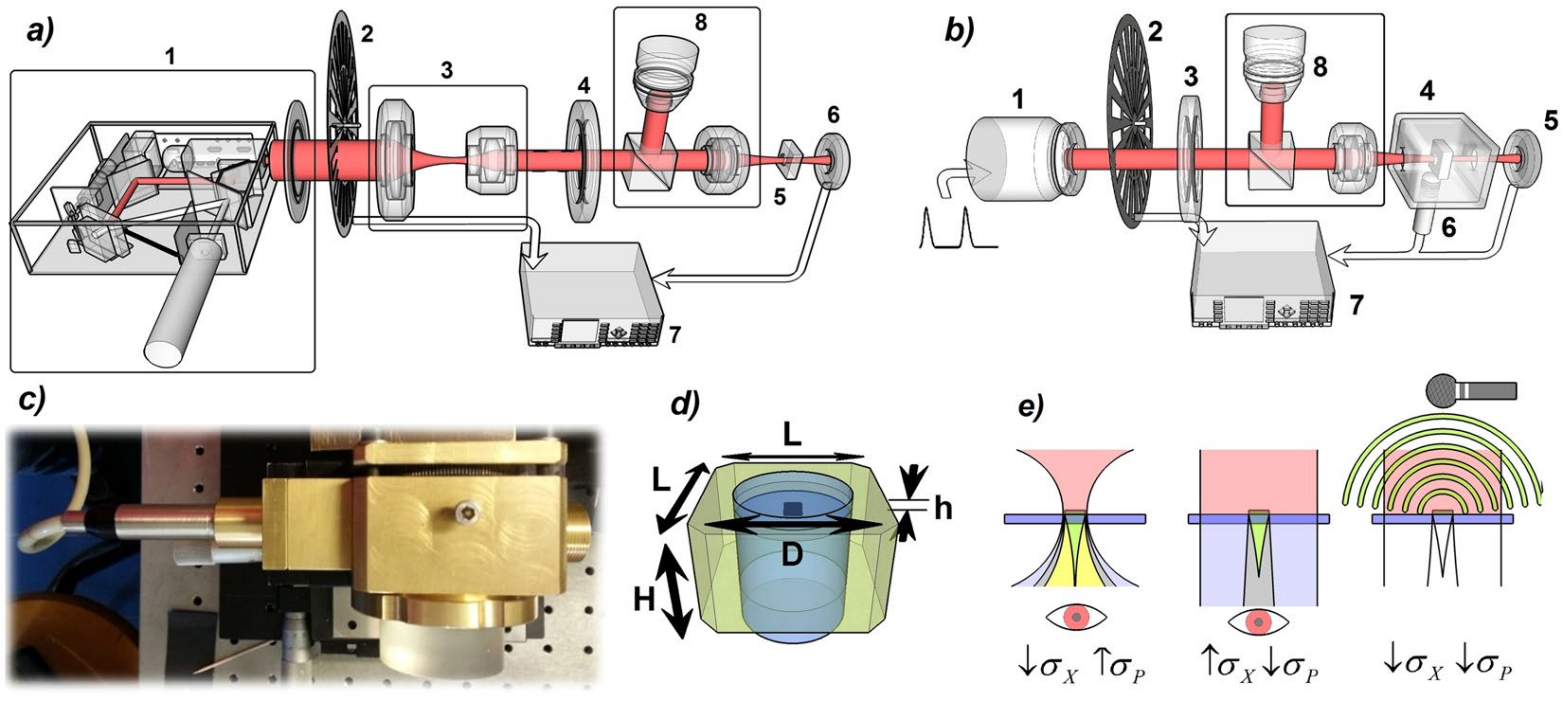
(**a**) Schematic view of the experimental setup for AO measurements. Here we display the polarized pump source (1), the chopper (2), the optic group of lenses (3) used to shrink the light source, the QWP (4), the sample (5) and the photodetector (6). A lock-in (7) control the signals and a set composed of a beam splitter and a microscope (8) is adopted to visualize and correctly aim the helices. (**b**) Schematic view of the experimental setup for PA measurements. We show the laser source (1), the chopper (2), the QWP (3), the PA chamber (4) containing the studied sample and the PD (6); the microphone (6) is partially installed inside the PA chamber; here again a lock-in (7) is connected to the main set-up. (**c**) PA chamber with Mic attached on its left side. (**d**) Schematic view of the main part of the PA chamber. L = 6 cm, H = 3,5 cm, D = 4 cm, h = 1 mm. Quartz glasses insulates the air chamber from external space. (**e**) Illustrative representation of a near-field optical (left), far-field optical (middle), and photoacoustic (right) measurement set-up. The colors associated to distinct zones refer to specific field characters: red = laser source; yellow = transmitted light through the helices with a high incidence angle; green = transmitted light through helices with normal incidence (desired data); grey = mixed field arising both from helices’ region and nude substrate region; light grey = light purely transmitted by naked substrate. This representation simplifies the reasons behind the adoption of PA in the place of more common optical approach: if we want to observe the field emerging purely from helices (green), we must located the observer in close proximity of the helices, and use high-precision confocal systems; an excessive focusing will add unwanted plane components (high *σ*_*P*_) to the measured field (yellow); a far-field optical set-up is easier to use, but it rather detects a mixed field (high *σ*_*X*_) predominantly coming from the substrate (grey), and the resulting CD will be largely suppressed. The adoption of PA allows to detect the pure helices’ footprint without the need of sophisticated systems like that of first AO set-up, still achieving the same precision and reliability.

For AO and PA measurements we assembled two distinct experimental setups (Fig. 2), both controlled by a personal computer (PC). For AO measurements (Fig. 2a) incident light coming from a Xenon arc lamp coupled to a monochromator passes through a linear polarizer and a wide-spectrum quarter wave plate (QWP), namely a Thorlabs AQWP05M-600 and a Thorlabs AQWP05M-980 for the λ ranges of (400–800) nm and (690–1200) nm, respectively. Incident light is focused onto the sample by means of 25X microscope objective, then detected by a photodetector (PD). Incident light is mechanically chopped at a frequency of 25 Hz used as reference for a lock-in amplifier.

The PA chamber is a brass container with a central circular cavity filled with quartz, with the exception of a small 1mm thick region filled with air and containing the sample (see Fig. 2c,d). The chamber features transparent apertures facing the incoming signals and the PD, plus a side channel for the Mic insertion, with plastic seals to grant a good acoustic insulation from the external environment. The modulation, operated by the chopper, has been added in order to recognize unwanted noises and spurious contributions, with its frequency and phase being used as markers for the main signals. Together with the PD’s, its control signals were monitored by a lock-in connected with the main PC. The sensitive microphone (Mic), used to ensure a clear record of the sound footprints of the optically absorbed power, was a Brüel & Kjær ANSI Type-4166^25^ combined with a Brüel & Kjær Pre-Amplifier Type-2619. Since the Mic-PreAmplifier system has its best signal-to-noise ratio (SNR) across 25 Hz, we set the modulation frequency to this value for the PA case.

## Experimental Results

The main advantage of measuring PA signals resides in their purity in terms of unwanted field contributions coming from the nude substrate. This is graphically summarized in Fig. 2e. AO measurements can be performed either with tightly focused light (left side) or with slightly focused light (centre); while with the first choice one sacrifices the quality of the injected field (high *σ*_*P*_), since high transverse momentum components are transmitted through the helices, in the second case a larger area is detected with respect to the helices’ set region, thus capturing unwanted signals from the substrate (high *σ*_*X*_). Therefore, reliable optical measurements on this kind of samples require the spatial selection of sample real image by using confocal set-up configurations^11^. As an alternative, PA measurements allow to overcome both these limitations, because they are based on the acoustic signal arising exclusively from the absorbing helices.

**Figure 3:**
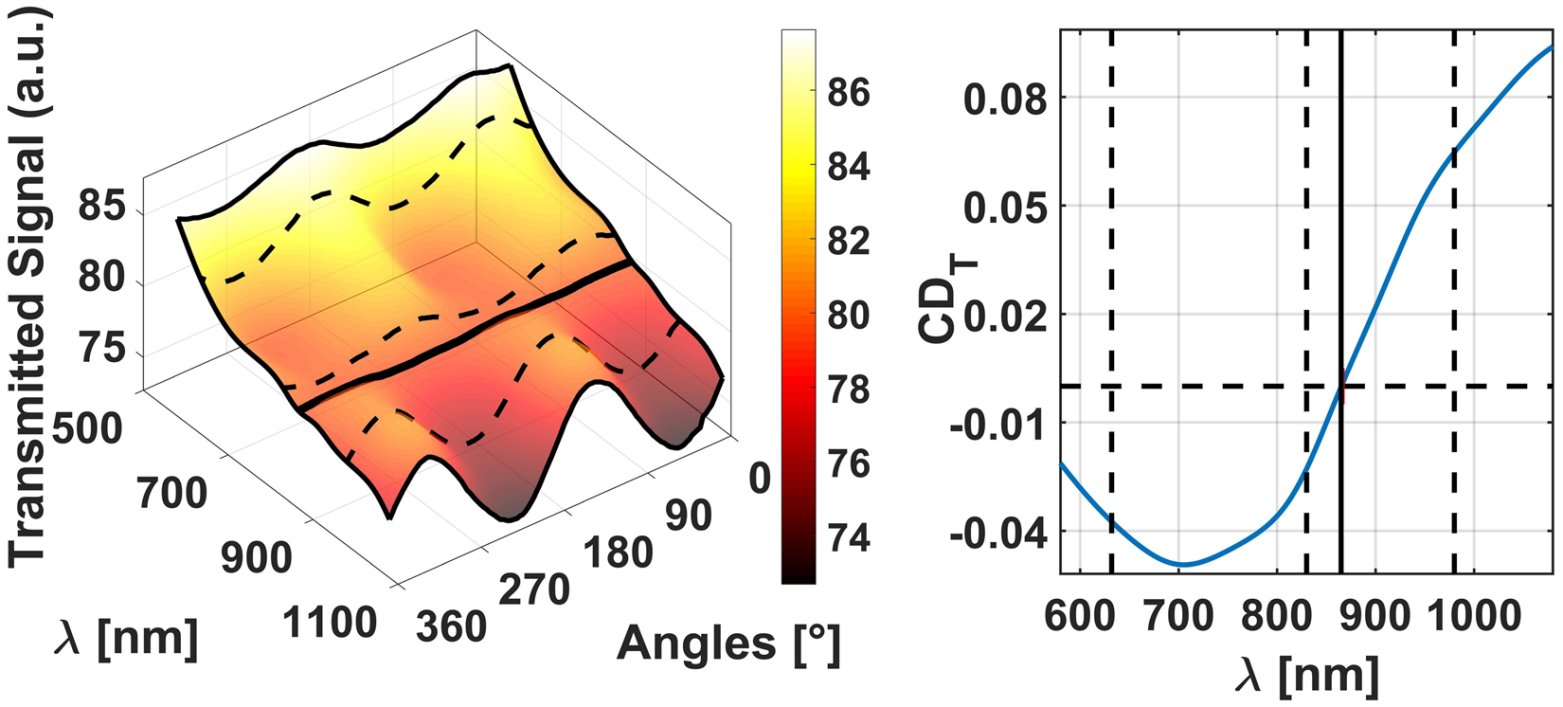
(**a**) Transmitted signal values (normalized for a common scaling factor) as a function of the vacuum wavelength λ and QWP orientation angle. (**b**) *CD*_*T*_ data extracted from (**a**) for the same wavelength range. We marked with dashed lines three wavelengths where we subsequently inspected the sample by means of the PA technique.

The results of the AO measurements have been assembled in a tridimensional function shown in Fig. 3a, where we plot the modulation of (normalized) light intensity reaching the PD as a function of the wavelength (λ) and of the QWP orientation angle. We estimate the average power impinging on the sample to be of the order of 1 mW, while the PD registered signals of the order of 500μV; the z-axis has been rescaled to a common reference for the sake of simplicity since we are only interested in the ratio between opposite circular polarizations. From the 3D plot of Fig. 3a we extrapolated the circular dichroism data in transmission (CD_T_) of Fig. 3b, after defining^26, 27^:

1 $$C{D}_{T}=2\cdot ({I}_{R}-{I}_{L})/({I}_{R}+{I}_{L})$$

The linear transmission regime is well recognizable by the perfectly sinusoidal shape of the transmitted light intensity spectra at a fixed wavelength. As derived in the Supplementary Information section, the square modulus of the total transmitted field T(*ω*, *z*) can be represented as follows:

2 $$T(\omega ,z)=\alpha (\omega ,z){T}_{H+S}(\omega )+\beta (\omega ,z)\sqrt{{T}_{H+S}(\omega )}+\gamma (\omega ,z)$$

where *T*_*H+S*_ is the transmission of the helices + substrate system and the 3 coefficients α, β and γ are dispersive functions dependent on the diffraction of light and, with the exception of α, also on the pure substrate’s transmittance, along with the vertical (z) position. Consequently, the *CD*_*T*_ profile measured in our experimental conditions is altered from the one belonging to the real helices-substrate system. The crossing point position between the LCP and RCP transmission curves (where *CD*_*T*_ = *0*) at λ = 856 nm is independent from diffraction effects since they affect at the same levels both the opposite circular polarizations, so it is a good reference for the experiments, which is expected at the same position also in PA measurements.

For the PA characterization we used four different laser sources, more precisely a 10 mW CW Coherent Compass 315 m green laser for λ = 532 nm, a 5 mW He-Ne laser for λ = 633 nm, and two different 10 mW Laser Diodes for λ = 830 nm and λ = 980 nm. The relative gaussian beam’s diameter at the output were 0.6 mm, 0.6 mm, 0.5 mm × 0.1 mm (elliptical) and 3.5 mm × 1.7 mm (elliptical), respectively. The sample has been illuminated from the helices side.

**Figure 4:**
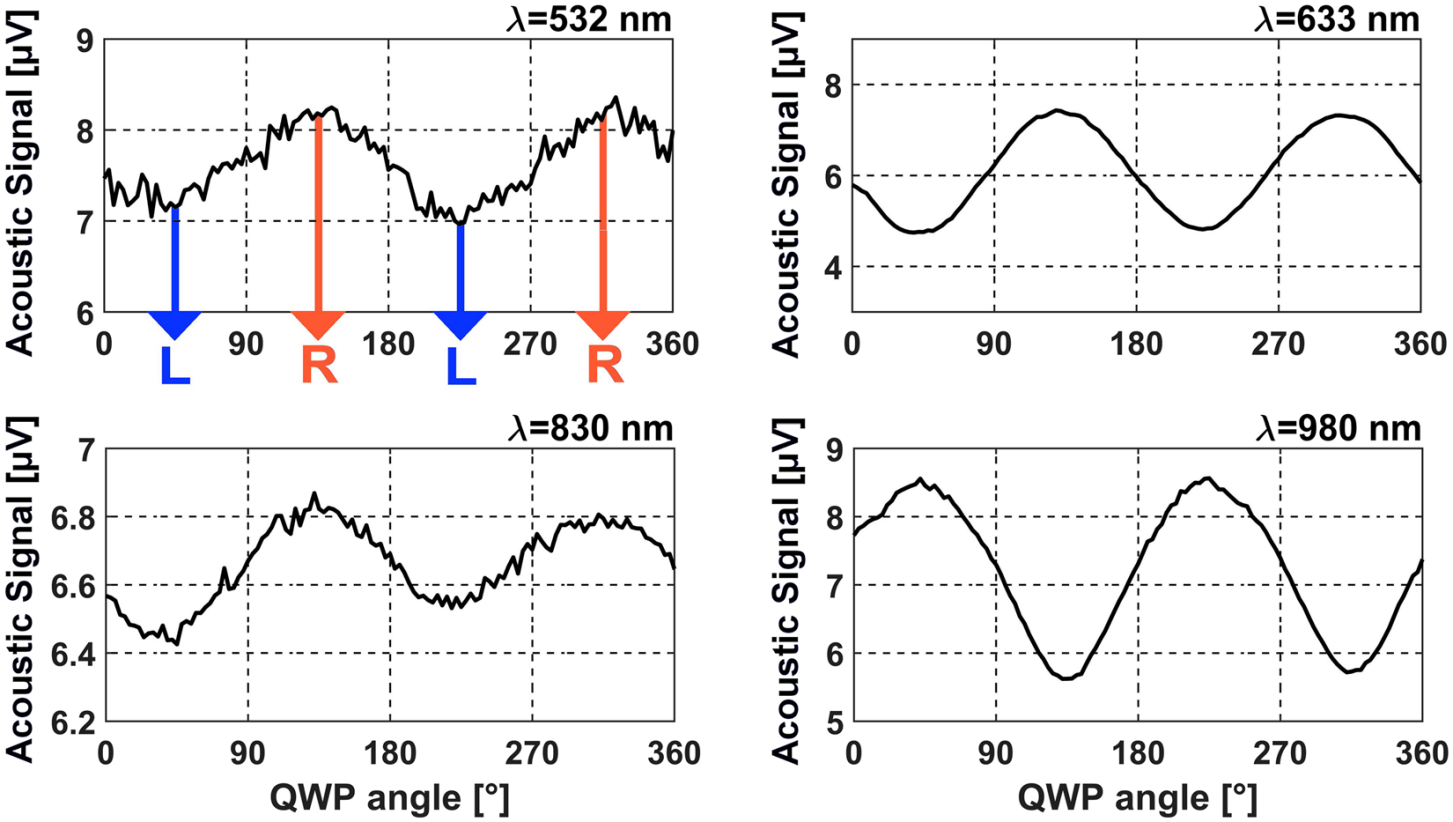
PA signal (in μV) as a function of the QWP orientation angle for four optical pumps; these spectra have been obtained under downward illumination. Extrapolated CD_A_ values are + 0.14 @ λ = 532 nm, + 0.41 @ λ = 633 nm, +0.1 @ λ = 830 nm, and −0.35 @ λ = 980 nm. The sound modulation was set at 25 Hz, and the measurements were performed with a detail of 2 degrees and a time step of 5 seconds between each measurement.

CD_A_ values arise exclusively from the absorbing helices, so the corresponding *β* and *γ* values of (2) are now exactly null. To appreciate the inherent CD of the measurements, we defined a coefficient related to absorbance^28^:

3 $$C{D}_{A}=2\cdot ({A}_{R}-{A}_{L})/({A}_{R}+{A}_{L})$$

which can be directly calculated from the measured PA signals (see Fig. 4).

For the first two lasers a chopper was physically part of the experimental apparatus, while for the two diodes it was replaced by a Newport Model-560 Laser Driver connected to their input port and controlled by the PC. Looking at Figs 3 and 4, we notice that the sign of *CD*_*A*_ is naturally reversed with respect to *CD*_*T*_’s. This comes from obvious reasons, considering that when the sample presents the maximum transmission (high T value) it usually presents the minimum absorption (low PA signal) and vice versa, and that a generic dichroic layer the same reflectance for the two opposite CP senses^29, 30^.

Moreover, by comparing AO and PA experiments, the differences between CDT and CDA levels, at λ = 633 nm, λ = 830 nm and λ = 980 nm, could be related to the scattering and diffraction contributions in the transmission measurements which are absent in the PA ones. This effect allows to identify absorption and diffraction effects in the transmission measurements since photoacoustics is intrinsically insensitive to light scattering.

## Numerical simulations

Aiming at having a more complete vision of the physical phenomenon, a detailed simulation of the entire optical process has been performed. We extensively used the Finite Difference Time Domain approach (FDTD, from Lumerical) for all the simulations.

The study of the helix permittivity has been carried out considering the compositional analysis data for this kind of FIBID nanostructures presented in ref. 12. According to the analysis, the nanohelices are composed by Pt clusters embedded in a carbon matrix (Pt percentage around 55%, C percentage around 40%, with a small amount of residual gallium coming from ion implantation). Pt is present as irregular elliptical globes with average diameter of 8 nm, forming clusters with quite homogeneous distribution along the entire helix filament (see Fig. S2 of the Supplementary Information). The carbon acts as a host in amorphous configuration, in good agreement with literature^31^.

Due to the relatively high percentage of inclusions, the Maxwell-Garnett and Brüggeman^32–34^ models cannot be considered acceptable to correctly evaluate the effective permittivities of the composite, so we add a further numerical step by performing a long series of simulations to emulate the random arrangement of Pt clusters in the carbon matrix. For each simulation, Pt clusters featured random positions and generic ellipsoidal shapes inside a C host, limiting the ellipsoids’ side lengths to the values before mentioned and keeping fixed the materials’ percentages according to the declared chemical composition. See ref. 12 for further details.

The Pt and C dielectric functions were extracted from Palik^35^, Djurišić & Li^36^, respectively. We included a composite material with C as the host medium and containing Pt ellipsoids into a FDTD box with a z extension of 1μm and large 0.5μm x 0.5μm on the Oxy plane, with couples of periodic boundary conditions at the Oxz and Oyz sides to emulate an infinite layer of homogeneous composite material. We illuminated the FDTD box with a vertical plane wave assuming a random arrangement of ellipsoids, and we extracted the composite material dispersion shown in Fig. S2.

We used these spectra to perform a second stage of simulations, where we investigated the behavior of a periodic distribution of single-wired helices located upon the multilayered substrate and featuring a certain variance among their geometrical parameters under downward illumination, using the geometrical parameters of the sample shown in Fig. 1, including random variations to emulate the fabrication tolerances: VP was set to 300 ± 10 nm, WD to 100 ± 10 nm and ED to 300 ± 20 nm, with ± indicating the standard deviation. In addition, we assumed a description formula for the helix central line given by:

4 $$\begin{array}{rcl}x(\theta ) & = & \frac{ED-WD}{2}\cdot \,\cos (\theta ),\,y(\theta )=\frac{ED-WD}{2}\cdot \,\sin (\theta )\\ z(\theta ) & = & \sqrt[p]{(1+{{\theta }_{0}}^{p})1+{({\theta }_{0}/\theta )}^{p}}{H}_{Tot},\,\theta \in [0,6\pi ]\end{array}$$

with *θ*_*0*_ = 6π*(0.9 ± 0.06), *p* = 10 ± 0.7, and *H*_*Tot*_ = 3*VP*. This last expression allowed to emulate the helix vertical shrinking effect during the growth process along the third dimension (see ref. 10).

**Figure 5:**
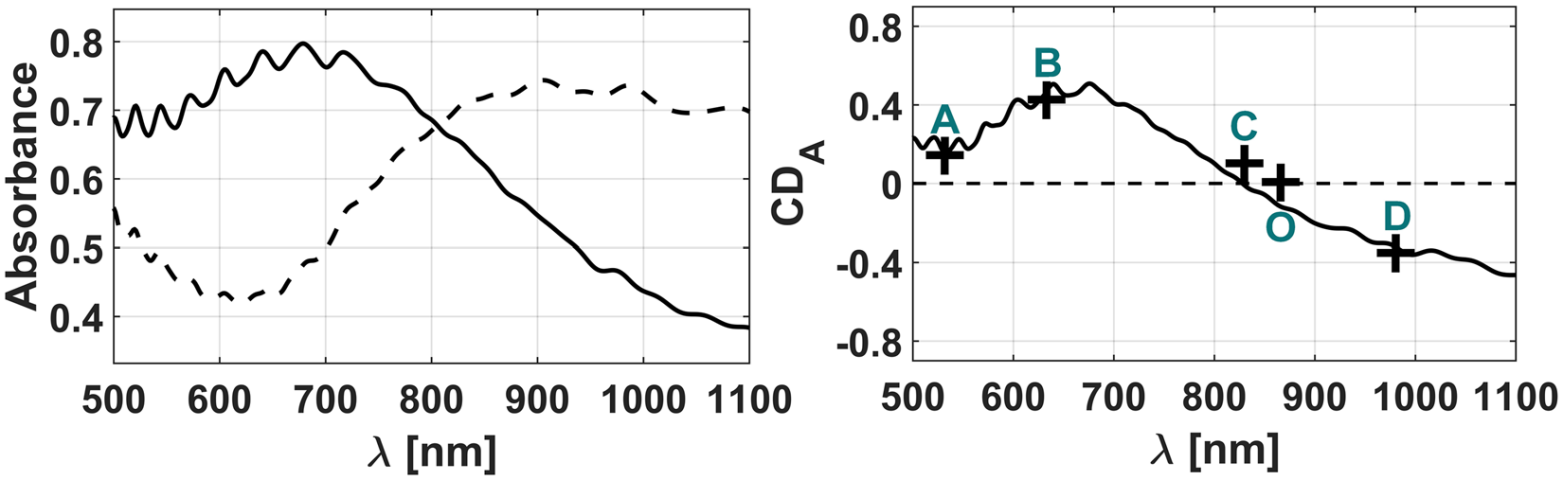
(**a**) Average absorption levels (dashed for Left CP and straight for Right CP) for periodic triple turn helices aligned with LP = 700 nm, and featuring a central line described by the equations in (4), with VP = 300 ± 10 nm, WD = 110 ± 10 nm, ED = 300 ± 20 nm, *θ*_*0*_ = 6π*(0.9 ± 0.06), *p* = 10 ± 0.7. (**b**) Corresponding CD_A_ spectrum as defined in (3); {A, B, C, D} indicate the *CD*_*A*_ values measured by means of the PA technique, while O indicates the AO crossing point.

Each single simulation was performed assuming all the helices to be identical by setting again periodic boundary conditions at the Oxz and Oyz sides, and provided the single transmittances and reflectances needed to get the simulated absorption levels for the two circularly polarized incident beams shown in Fig. 5a. These data have been then employed for calculating CD_A_ shown in Fig. 5b, along with experimental data referred to the single wavelengths of 532 nm, 633 nm, 830 nm, and 980 nm (indicated as points A, B, C and D, respectively). We also calculated the effective refractive indexes^30^ associated to the helices layer surrounded by vacuum for the two senses of circular polarization, as shown in Fig. S3 of the Supplementary Information. The helices appear to resonate approximately at 261 THz (λ = 1150 nm) and 441 THz (λ = 680 nm) under normal plane incidence with left and right circular polarization, respectively.

The simulated spectra demonstrated to be in good agreement with the main experimental data, as can be seen by the close proximity of the [A,B,C,D,O] points from the straight line in the graph. Small discrepancies arise potentially due to negligible differences between the adopted dispersion curves and the ones related to the real composite material, and to fabrication tolerances in the helices geometry. Looking at Fig. 3b and 5b, we note also a small difference between the AO and PA crossing points, mainly due to small anisotropies on the experiment which naturally leads to spurious dichroism; this latter sums up with the real intrinsic dichroic of the helices’ ensemble^12^ and that of the substrate hosting the helices. A more detailed interpretation about this shift, which however is sufficiently small to be negligible, can be read in the Supplementary Information. As demonstrated by experimental measurements^11, 12^, the helices respond to the incoming electromagnetic field with low reflection levels and a good impedance match, acting as a wide band effective filter for circular dichroism both for the transmitted fields and the absorbed power.

## Conclusions

We investigated the dichroic character of a nano-structured sample composed of an ordered ensemble of nano-sized helices fabricated by FIBID process built on a small area. We have optically characterized the nanohelix array by AO and PA techniques. By comparing optical and photoacoustic techniques, AO measurements are affected by high diffraction effects and, in addition, in the case of reduced sample area, empty area transmission hinders the purity of fields, while, PA measurement has allowed to obtain the pure and precise CD of the chiral sample by measuring scattering independent absorptions solely related to the helices.

An accurate numerical study allowed to perform a complete analysis of the electromagnetic phenomenon, confirming the experimental results. While the reduction of the array size helps to decrease fabrication time and eases placement inside integrated systems, it usually compromises CD detection, but thanks to the PA technique we have overcome this limitation. Since we obtained high CD values over a very large bandwidth for this sample, as per nature of this class of materials, we consider it a suitable candidate for innovative optical filters operating in the NIR, VIS and potentially UV frequency regions.

## Electronic supplementary material

Supplementary Information

## References

[1] Nordén, B., Rodger, A., Dafforn, T. *Linear Dichroism and Circular Dichroism: A Textbook on Polarized-Light Spectroscopy (Special Publication), 1st Edition* (Society of Chemistry, 2010).

[2] Bertolotti M, Belardini A, Benedetti A, Sibilia C (2015). Second harmonic circular dichroism by self-assembled metasurfaces [Invited]. JOSA B.

[3] Balanis, C. A. Antenna Theory - Analysis and Design, 3rd Edition (John Wiley & Sons, 2005).

[4] Gansel JK (2009). Gold helix photonic metamaterial as broadband circular polarizer. Science.

[5] Xiong Z (2012). Assembling optically active and nonactive metamaterials with chiral units. AIP Advances.

[6] Yang Z (2012). Polarization properties in helical metamaterials, Front. Of Optoelec..

[7] Kaschke J, Wegener M (2015). Gold triple-helix mid-infrared metamaterial by STED-inspired laser lithography. Opt. Lett..

[8] Behera S (2015). Joseph, N-single-helix photonic-metamaterial based broadband optical range circular polarizer by induced phase lags between helices. J. App. Opt..

[9] Mark AG, Gibbs JB, Lee T-C, Fisher P (2013). Hybrid nanocolloids with programmed three-dimensional shape and material composition. Nature Mat..

[10] Esposito M (2014). Three Dimensional Chiral Metamaterial Nanospirals in the Visible Range by Vertically Compensated Focused Ion Beam Induced-Deposition. Adv. Opt. Mat..

[11] Esposito M (2015). Nanoscale 3D Chiral Plasmonic Helices with Circular Dichroism at Visible Frequencies. ACS Photonics.

[12] Esposito M (2015). Tailoring chiro-optical effects by helical nanowire arrangement. Nanoscale.

[13] Tasco V (2016). Three-dimensional nanohelices for chiral photonics. Appl. Phys. A.

[14] Esposito M (2015). Triple-helical nanowires by tomographic rotatory growth for chiral photonics. Nat. Comm..

[15] Mandelis, A. Principles and Perspectives of Photothermal and Photoacoustic Phenomena in *Progress in Photothermal and Photoacoustic Science and Technology, Vol 1* (Appleton & Lange, 1991).

[16] Wang, L. V. *Photoacoustics Imaging and spectroscopy* (CRC Press, 2009).

[17] Saxe JD, Faulkner TR, Richardson FS (1979). Photoacoustic detection of circular dichroism. Chem. Phys. Lett..

[18] Garcia ME, Brouder Ch, Bennemann KH (1997). Theory for the photoacoustic response to circularly polarized X-rays: How to detect magnetism by using a microphone. Solid. State Comm..

[19] Uversky V. N., Permyakov, E. A. *Methods in Protein Structure and Stability Analysis* **(**Nova Science Publishers, 2007).

[20] Palmer, R. A., Roark, J. C., Robinson, J. C. Photoacoustic detection of natural circular dichroism in crystalline transition metal complexes in *Stereochemistry of Optically Active* Transition Metal *Compounds* (ed. Douglas, E. B., Saito, Y.) *ACS Symposium Series* **119** 20, 375–395 (1980).

[21] Matsuda O, Larciprete MC, Li Voti R, Wright O (2015). Fundamentals of picosecond laser ultrasonics. Ultrasonics.

[22] Belardini A (2016). Chiral light intrinsically couples to extrinsic/pseudo-chiral metasurfaces made of tilted gold nanowires. Sci. Rep..

[23] Zelewski S (2016). Photoacoustic spectroscopy of absorption edge for GaAsBi/GaAs nanowires grown on Si substrate. Appl. Phys. Lett.

[24] Esposito M (2016). Programmable Extreme Chirality in the Visible by Helix-Shaped Metamaterial Platform. Nano Lett..

[25] Brüel & Kjær, Product Data: Condenser Microphone Cartridges - Types 4133 to 4181, http://www.bksv.com/doc/Bp0100.pdf (Date of access:01/03/2017).

[26] Dorman BP, Hearst JE, Maestre MF (1973). UV absorption and circular dichroism measurements on light scattering biological specimens. Methods Enzymol..

[27] Benedetti A, Belardini A, Veroli A, Centini M, Sibilia C (2014). Numerical tailoring of linear response from plasmonic nano-resonators grown on a layer of polystyrene spheres. Jour. of App. Phys..

[28] Ferenc Zsila MD (2010). Electronic Circular Dichroism Spectroscopy. Pharm. Sci. Enc..

[29] Landau, L. D., Lifshitz, E. M. *Electrodynamics of Continuous Media, Vol.8 of the Course of Theoretical Physics* **(**Pergamon Press, 1960).

[30] Castanié A, Mercier J-F, Félix S, Maurel A (2014). Generalized method for retrieving effective parameters of anisotropic metamaterials. Opt. Expr..

[31] De Teresa JM (2009). Origin of the Difference in the Resistivity of As-Grown Focused-Ion- and Focused-Electron-Beam-Induced Pt Nanodeposits. Jour. of Nanomat..

[32] Weiglhofer WS, Lakhtakia A, Michel B (1997). Maxwell Garnett and Bruggeman formalisms for a particulate composite with bianisotropic host medium. Microw. Opt. Technol. Lett..

[33] Tuck, C. *Effective Medium Theory 1st Ed*. (Oxford University Press, 1999).

[34] Weiglhofer, W. S., Lakhtakia, A. *Introduction to Complex Mediums for Optics and Electromagnetics* (SPIE Press, Bellingham, Wash, USA, 2003).

[35] Palik, E. D. *Handbook of Optical Constants of Solids* **(**Academic Press, 1998).

[36] Djurišić AB, Li EH (1999). Optical properties of graphite. Jour. of App. Phys..

