# Supplementary material for: Precise detection of circular dichroism in a cluster of nano-helices by photoacoustic measurements
Source: Sci Rep. 2017 Jul 12;7:5257. doi: 10.1038/s41598-017-05193-4 (PMC5507913; doi:10.1038/s41598-017-05193-4)
Supplement: Supplementary file 1 — Supplementary Information [file 41598_2017_5193_MOESM1_ESM.pdf]

Supplementary Information

Precise detection of circular dichroism in a cluster of nano-helices by photoacoustic measurements.

Alessio Benedetti*[1,2], Badrul Alam[1,2], Marco Esposito[3,4], Vittorianna Tasco[3], Grigore Leahu[1], Alessandro Belardini[1], Roberto Li Voti[1], Adriana Passaseo[3], Concita Sibilia[1,3]

1 S.B.A.I. Department, Physics Section, "Sapienza: Università di Roma", Via A. Scarpa14/16, I-00161 Rome (Italy).

2 D.I.E.T. Department , "Sapienza: Università di Roma", Via Eudossiana 18, I-00184 Rome (Italy).

3 CNR Nanotec, Istituto di Nanotecnologia, Polo di Nanotecnologia, c/o Campus Ecotekne, via Monteroni I-73100 Lecce (Italy).

4 "Università del Salento", Dip. Mat-Fis "Ennio De Giorgi", Via Arnesano I-73100 Lecce (Italy).

** alessio.benedetti@uniroma1.it*

1) Permittivity function of the filament composing the helix, and helices' layer spectral properties.

In order to perform all the 3D simulations of the helixes, we firstly needed to know the internal filament refractive index spectrum. We managed this stage by means of an effective refractive index approach.

The effective refractive indexes employed in the simulations take into account the filament internal composition already described in Ref.[1]. The filament is composed of large clusters of tiny Pt and Ga spheroids located inside a homogeneous amorphous carbon host environment. The Pt and Ga inclusions' percentages are estimated to be around 55% and 5%, approximatively, with the reaming part of volume occupied by the host C. The large average diameter of the spheroids (ranging from 6 to 12nm both for Pt and Ga) suggests that setting the canonical refractive indexes spectra is still a good assumption and that an evaluation of the e.m. properties by means of an effective medium approximation seemed to be possible, so we enclosed a random arrangement of spheroids in a large FDTD box with periodic boundary conditions (PBC) and illuminated this system with a plane wave with normal incidence (as shown in fig. S1).

A sufficiently large extension is set to suppress any spurious reflection coming from the opposite termination of the FDTD box, and a monitor collects the complex values of reflected field for the wavelengths of interest (approximately [0.3 μm -1.6 μm]). After some numerical passages, we retrieved the reflection coefficients of the overall compounds, and we finally calculated the required refractive indexes by taking advantage of the following formula:

$$n(\lambda) = n_{Host}(\lambda) \cdot \frac{1-r(\lambda)}{1+r(\lambda)}$$

where n_{Host} is the host refractive index and r the complex reflection coefficient.

where n_{Host} is the host (air) refractive index and r the complex reflection coefficient.

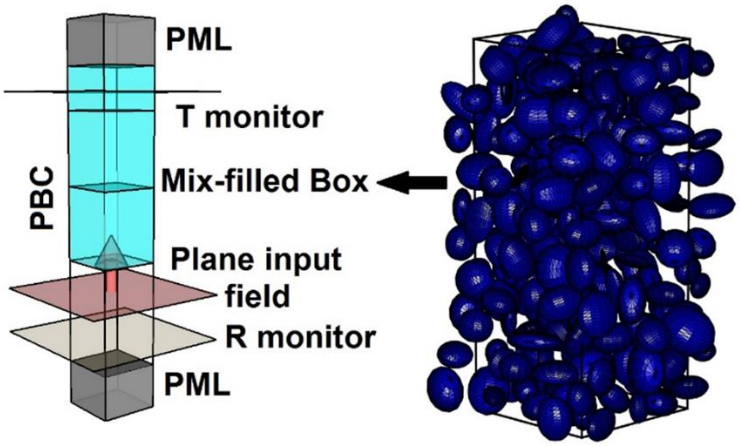

Figure S1. On the left, schematic view of the entire FDTD system. A linearly polarized plane wave vertically directed toward the top impinges on stacked boxes filled with the mixture. An example of mixture is displayed on the right, with a 40x40x100nm box filled by the inclusions, and whose background index is set to the carbons. The spheroids in the picture feature an average diameter of 8nm, and they randomly cover 30% of the total volume.

The Pt, Ga and C spectra used for this purpose are shown in the top line of fig.S2. Thanks to the numerical calculations, we obtained the final index spectrum shown in the bottom graph of fig.S2. The final mixture presents a strong metal character, which suggests the presence of surface plasmon polaritons along the helixes' bodies when they are illuminated.

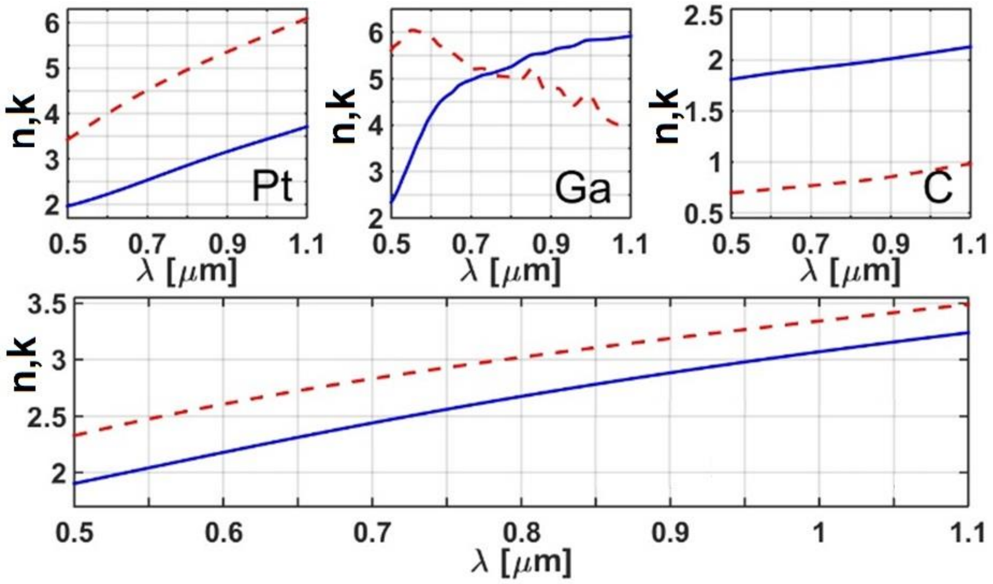

Figure S2. (Above) the dispersion spectra adopted for the 3 materials and (below) the resulting dispersion adopted for the filament's material, calculated with the random cluster simulation technique. Real indexes are plotted in blue straight lines, while red dashed lines refer to imaginary indexes.

We adopted a similar procedure to calculate the effective phase index for a virtual layer composed of helices floating in the vacuum. We calculated the complex reflection and transmission coefficients of the helixes layer and, together with the assumption of an effective thickness of 950nm, we calculated the effective refractive index of an equivalent homogeneous slab layer [2], whose profile we show in fig. S3.

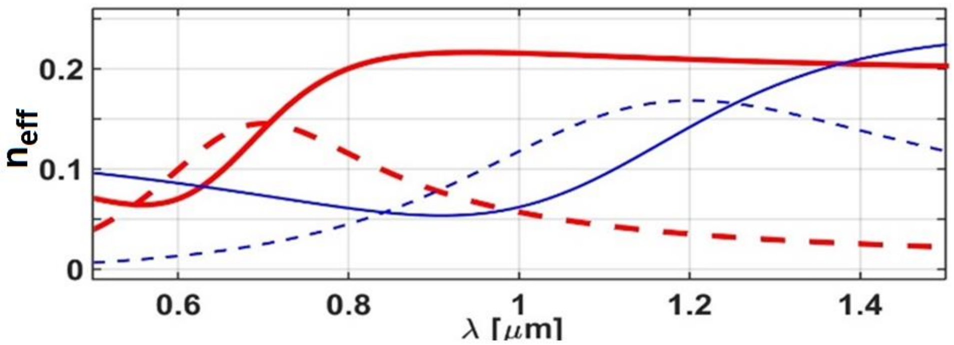

Figure S3. Effective refractive index spectra for the opposite sense of polarization. Straight (dotted) lines refer to the real (imaginary) part. Red-thick (blue-thin) lines refer to the right (left) circular polarization.

The spectra are different for the two signs of circular polarization. Indeed, these indexes correspond to relative permittivities which present the typical Drude-Lorentz resonant profile:

$$n^{L/R}(\omega) = \sqrt{\varepsilon_r^{L/R}(\omega)} = \sqrt{\varepsilon_\infty - \frac{\left(\omega_P^{L/R}\right)^2}{\omega^2 - \left(\omega_0^{L/R}\right)^2 - \iota\omega\gamma^{L/R}}} \tag{5}$$

with *L* and *R* indicating the left and right circular polarization, and ε_r the relative permittivity. Good fit parameter values are ε_∞ =0.12, $\omega^L{}_P$=4.9415·10^{14}, $\omega^R{}_P$=8.0667·10^{14}, $\omega^L{}_0$=17.4707·10^{14}, $\omega^R{}_0$=28.5203·10^{14}, γ^L=8.1772·10^{14}, γ^R=15.5254·10^{14}. Consequently, the helices appear to resonate approximately at 261THz and 441THz under normal plane incidence with left and right circular polarization, respectively.

The simulations for the absorption spectra have included also the presence of the substrate, which is composed of a multilayered structure (see fig.1). The refractive index spectra adopted for the 4 involved materials are summarized in the plots of fig.S4. We used dispersions retrieved from Barker & Ilegems (ordinary axis, 1973) [3] for the GaN layer, from Pastrňák & Roskovcová (ordinary axis, 1966) [4] for the AlN layer, from Malitson &Dodge (synthetic sapphire, ordinary axis, 1972) [5] for the Al_2O_3 substrate, and our own measurements for the Al0.3Ga0.7N.

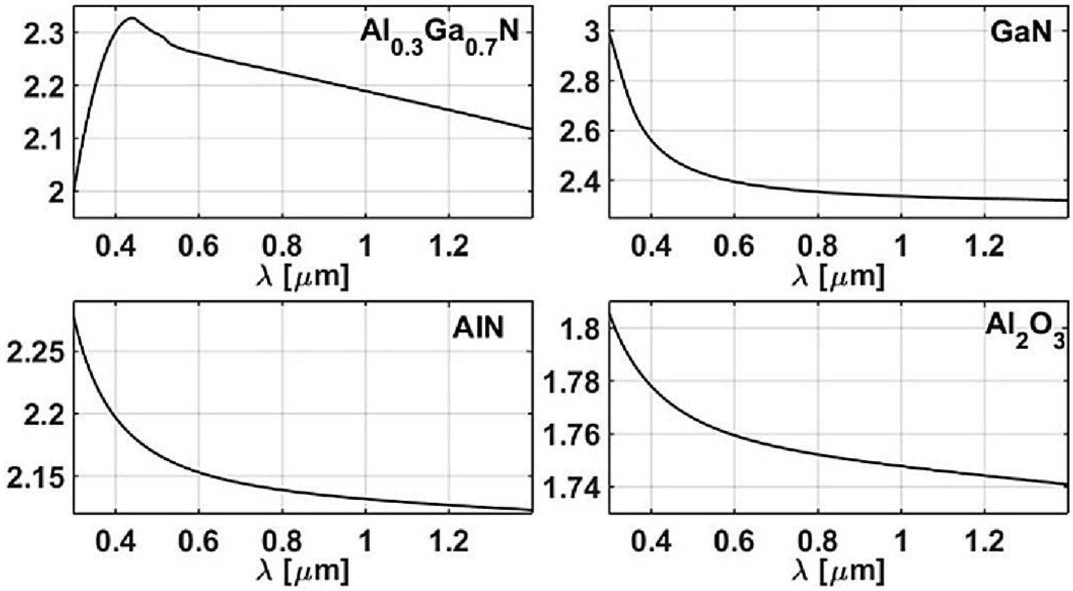

Figure S4. Refractive index spectra for the four materials compounding the multilayered substrate. The materials are perfectly lossless in the observed wavelength region. See fig.1 for the substrate internal structure.

2) Crossing point shifts and anisotropy effects.

Small anisotropies on the experiment naturally leads to spurious dichroism that sums up with the real intrinsic dichroic character of the helices' ensemble; these anisotropies are both related to the helices [6] and the hosting substrate. These secondary dichroisms leads to a weak shift of few nanometers for the two circular polarization absorption crossing point (i.e., the wavelength at which the total dichroism is null), and at analytical level to the break of proportionality between the CD related to transmittance (CD_T), on one hand, and the CD related to absorbance (CD_A), on the other hand.

Indeed, we know that under ideal conditions, CD_T and CD_A must be proportional (with a dispersive coefficient):

$$CD_A = 2\frac{A_R - A_L}{A_R + A_L} = 2\frac{T_L - T_R}{2\cdot(1-R)-(T_R+T_L)} = -CD_T \cdot \frac{1}{\frac{2\cdot(1-R)}{T_R+T_L}-1} = -CD_T \cdot \frac{1}{\frac{1-R}{T_{Lin}}-1} = -CD_T \cdot \frac{T_{Lin}}{A_{Lin}} \tag{6}$$

where R is the common reflectance for the two polarizations [2], where A_{Lin} and T_{Lin} refers to the absorbed and transmitted spectra under linear polarization. In presence of anisotropies, the incidence (and consequent partial absorption) of a left/right circular polarization gives rise also to the transmission of polarizations with opposite circular sign:

$$CD_A = 2\frac{A_R - A_L}{A_R + A_L} \approx 2\frac{T_L - T_R + T_{R,L} - T_{L,R}}{2\cdot(1-R)-(T_R+T_L)} = \left(-CD_T + \frac{T_{R,L} - T_{L,R}}{T_{Lin}}\right)\cdot \frac{1}{\frac{1-R}{T_{Lin}}-1} \tag{7}$$

with $T_{R,L}$ ($T_{L,R}$) being the transmittance of right (left) circular polarization promoted by the incidence of left (right) circular input field.

3) Diffraction effects in Circular Dichroism.

The diffraction properties of light naturally contribute to deform the CD detected in transmission and to a second shift of the crossing point along the spectrum. To better explain this fact, we take advantage of a scalar approach and to the paraxial approximation to characterize the output field observed across the central axis (x'=0 & y'=0) beneath the helices at different heights as the superposition of two distinct contributions, one coming from the vertical stack of helices and substrate, and one coming from the naked substrate. Assuming that no anisotropies take place in this instance, and approximating the input field to an infinite plane wave, this output field is estimated to be:

$$E_O(z) \approx E_I \cdot \left\{ t_{H+S}(\omega) \cdot \left[e^{\iota\kappa_0 z} \cdot \iint_{S_H} e^{+\iota\frac{\kappa_0}{2z}\left(x'^2+y'^2\right)} dx'dy' \right] + \right. \tag{8a}$$

$$\left. + \left[t_S(\omega) \cdot e^{\iota\kappa_0 z} \cdot \iint_{\mathbb{R}^2 \setminus S_H} e^{+\iota\frac{\kappa_0}{2z}\left(x'^2+y'^2\right)} dx'dy' \right] \right\} = E_I \cdot \left[c_1(\omega,z) \cdot t_{H+S}(\omega) + c_2(\omega,z) \right]$$

where E_I is the input electric field, S_H is the sample surface covered by helices, R^2-S_H is remaining unpatterned surface, and c_1 and c_2 are:

$$c_1(\omega,z) = e^{\iota\kappa_0 z} \cdot \iint_{S_H} e^{+\iota\frac{\kappa_0}{2z}\left(x'^2+y'^2\right)} dx'dy' \tag{8b}$$

$$c_2(\omega,z) = t_S(\omega) \cdot e^{\iota\kappa_0 z} \cdot \iint_{\mathbb{R}^2 \setminus S_H} e^{+\iota\frac{\kappa_0}{2z}\left(x'^2+y'^2\right)} dx'dy'$$

Consequently, the transmitted intensity will be:

$$\Pi_O(z) = \frac{\left|E_O(z)\right|^2}{2\zeta_0} \approx \frac{\left|E_I(z)\right|^2}{2\zeta_0} \cdot \left| c_1(\omega,z) \cdot t_{H+S}(\omega) + c_2(\omega,z) \right|^2 = \tag{9}$$

$$= \Pi_I \cdot \left| c_1(\omega,z) \cdot t_{H+S}(\omega) + c_2(\omega,z) \right|^2$$

So the transmittance will be a dispersive, z-dependent function:

$$T(\omega,z) = \frac{\Pi_O(z)}{\Pi_I} \approx \left| c_1(\omega,z) \cdot t_{H+S}(\omega) \right|^2 + 2 \cdot \mathrm{Re}\left[c_1(\omega,z) \cdot c_2^*(\omega,z) \cdot t_{H+S}(\omega) \right] + \left| c_2(\omega,z) \right|^2 = \tag{10}$$

$$= \left| c_1(\omega,z) \right|^2 \cdot T_{H+S}(\omega) + 2 \cdot \mathrm{Re}\left[c_1(\omega,z) \cdot c_2^*(\omega,z) \cdot e^{\iota\Phi_{t_{H+S}}(\omega)} \right] \cdot \left| t_{H+S}(\omega) \right| + \left| c_2(\omega,z) \right|^2 =$$

$$= \alpha(\omega) \cdot T_{H+S}(\omega) + \beta(\omega,z) \cdot \sqrt{T_{H+S}(\omega)} + \gamma(\omega,z)$$

having actuated the following replacements:

$$\alpha(\omega) = \left| c_1(\omega,z) \right|^2 = \left| e^{\iota\kappa_0 z} \cdot \iint_{S_H} e^{+\iota\frac{\kappa_0}{2z}\left(x'^2+y'^2\right)} dx'dy' \right|^2 \tag{11}$$

$$\beta(\omega,z) = 2 \cdot \mathrm{Re}\left[c_1(\omega,z) \cdot c_2^*(\omega,z) \cdot e^{\iota\Phi_{t_{H+S}}(\omega)} \right]$$

$$\gamma(\omega,z) = \left| c_2(\omega,z) \right|^2 = \left| t_S(\omega) \cdot e^{\iota\kappa_0 z} \cdot \iint_{\mathbb{R}^2 \setminus S_H} e^{+\iota\frac{\kappa_0}{2z}\left(x'^2+y'^2\right)} dx'dy' \right|^2$$

Among β and γ, which represent the spurious contribution to the exact transmittance which is related only to the region of superposition of helices and substrate, the coefficient γ appears to be stronger and more influent to the increasing difference among CD_T and CD_A. Indeed, considering eq.(10a), we have:

$$CD_T = \frac{\alpha(\omega) \cdot \left[T_{H+S,Right}(\omega) - T_{H+S,Left}(\omega) \right] + \beta(\omega,z) \cdot \left[\sqrt{T_{H+S,Right}(\omega)} - \sqrt{T_{H+S,Left}(\omega)} \right]}{\alpha(\omega) \cdot \frac{T_{H+S,Right}(\omega) + T_{H+S,Left}(\omega)}{2} + \beta(\omega,z) \cdot \frac{\sqrt{T_{H+S,Right}(\omega)} + \sqrt{T_{H+S,Left}(\omega)}}{2} + \gamma(\omega,z)} =$$

$$= \frac{CD_{T,H+S} + \frac{\beta(\omega,z)\cdot\left[\sqrt{T_{H+S,Right}(\omega)} - \sqrt{T_{H+S,Left}(\omega)}\right]}{\alpha(\omega)\cdot\frac{T_{H+S,Right}(\omega)+T_{H+S,Left}(\omega)}{2}}}{1+\frac{\beta(\omega,z)\cdot\frac{\sqrt{T_{H+S,Right}(\omega)}+\sqrt{T_{H+S,Left}(\omega)}}{2}+\gamma(\omega,z)}{\alpha(\omega)\cdot\frac{T_{H+S,Right}(\omega)+T_{H+S,Left}(\omega)}{2}}} \quad (12)$$

and considering eq.6 we shall have:

$$CD_T = \frac{-CD_{A,H+S}\cdot\left(\frac{1-R_{H+S}}{\frac{T_{H+S,Right}(\omega)+T_{H+S,Left}(\omega)}{2}}-1\right)+\frac{\beta(\omega,z)\cdot\left[\sqrt{T_{H+S,Right}(\omega)}-\sqrt{T_{H+S,Left}(\omega)}\right]}{\alpha(\omega)\cdot\frac{T_{H+S,Right}(\omega)+T_{H+S,Left}(\omega)}{2}}}{1+\frac{\beta(\omega,z)\cdot\frac{\sqrt{T_{H+S,Right}(\omega)}+\sqrt{T_{H+S,Left}(\omega)}}{2}+\gamma(\omega,z)}{\alpha(\omega)\cdot\frac{T_{H+S,Right}(\omega)+T_{H+S,Left}(\omega)}{2}}} \quad (13)$$

The presence of the coefficients β and γ in the denominator at the right-hand side of eq.13 leads to an evident reduction of the measured CD_T values. Eq.6 explains also why any crossing point of absorption-related CD should be the same of transmittance-related CD (excluding anisotropies described In previous section), since the convergence of the two CP transmittances to a common value deletes both the term in the numerator at the last member of eq.13 and CD_T, thus implying the annihilation of CD_A.

4) Dichroic character dependence on illumination direction.

A second set of measurements, performed reversing the studied sample, revealed a certain dependence of the CD spectrum on the direction of illumination. We show the collective experimental data set in figure S5.

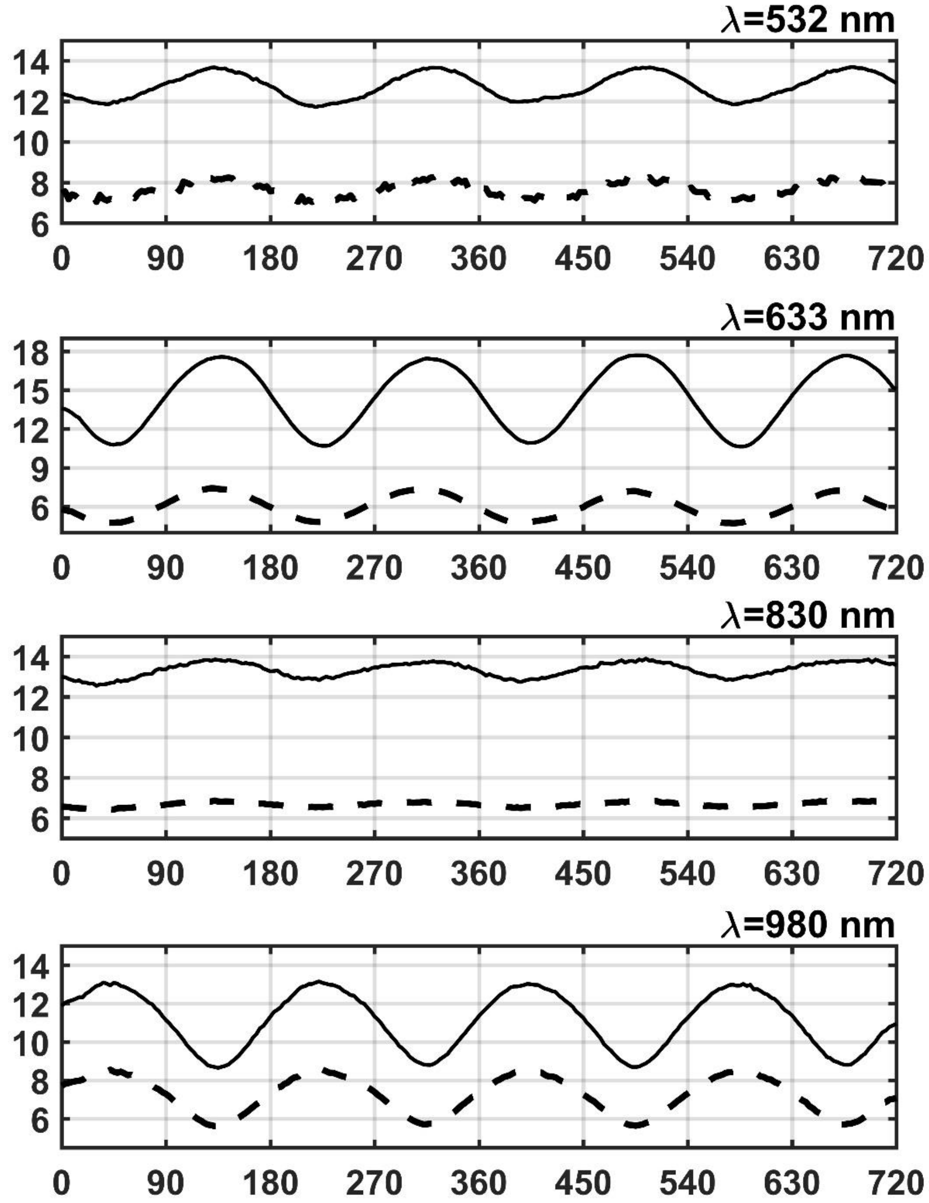

Figure S5. Acoustic signal as a function of the quarter wave phase plate for the 4 investigated wavelengths under upward and downward illumination, plotted in straight and dashed lines, respectively. The upward set of experimental data is the same plotted also on the main publication.

Despite showing extremely similar values, there were small percentage differences in the CD spectra (less than 10%) for the two sets of data related to the downward and upward illumination. These differences can be related to substrate effects on the measurements rather than real systematic errors, and in order to better understand this last effect, we performed a further simulation changing position and illumination direction of the source plane in the numerical setup.

To understand the origins of this discrepancy, we start stating that the absorption of two counterpropagating waves in a lossy layer is given by:

$$A = \varepsilon_0 \int_H \left\{ \int_S \mathrm{Im}\left[\varepsilon_r \left(\vec{r} \right) \right] \cdot \left\| \overrightarrow{E^{\uparrow}} \left(\vec{r} \right) e^{+\imath\beta_1 z} + \overrightarrow{E^{\downarrow}} \left(\vec{r} \right) e^{-\imath\beta_2 z} \right\|^2 dS \right\} dz =$$

$$= \varepsilon_0 \int_H \left\{ \int_S \mathrm{Im}\left[\varepsilon_r\left(\vec{r}\right)\right] \cdot \left[\left\| \overrightarrow{E^{\uparrow}}\left(\vec{r}\right) \right\|^2 e^{-2\mathrm{Im}(\beta_1)z} + \left\| \overrightarrow{E^{\downarrow}}\left(\vec{r}\right) \right\|^2 e^{+2\mathrm{Im}[\beta_2]z} \right] dS \right\} dz +$$

$$+ \varepsilon_0 \int_H \left\{ \int_S \mathrm{Im}\left[\varepsilon_r\left(\vec{r}\right)\right] \cdot \left[2\,\mathrm{Im}\left[\overrightarrow{E^{\uparrow}}\left(\vec{r}\right) \bullet \overrightarrow{E^{\downarrow *}}\left(\vec{r}\right) e^{+\iota\left(\beta_1 + \beta_2^*\right)z} \right] \right] dS \right\} dz \approx$$

$$\approx \varepsilon_0 \int_H \left\{ \int_S \mathrm{Im}\left[\varepsilon_r\left(\vec{r}\right)\right] \cdot \left[\left\| \overrightarrow{E^{\uparrow}}\left(\vec{r}\right) \right\|^2 e^{-2\mathrm{Im}(\beta_1)z} + \left\| \overrightarrow{E^{\downarrow}}\left(\vec{r}\right) \right\|^2 e^{+2\mathrm{Im}[\beta_2]z} \right] dS \right\} dz = A^{\uparrow} + A^{\downarrow} \tag{14}$$

with S and H being the surface and thickness of the lossy volume. Assuming that two counterpropagating waves are travelling along the helices layer, we calculate the absorption for downward and upward illumination truncating this process after the first reflection step:

$$A^{\downarrow}_{R/L} \approx A_{H,R/L} + \left(1 - A_{H,R/L} - R_H\right) \cdot \frac{R_{S,\downarrow}}{\left|1 - r_{S,\downarrow} r_H\right|^2} \cdot A_{H,L/R} \approx A_{H,R/L} + \left(1 - A_{H,R/L} - R_H\right) \cdot R_{S,\downarrow} \cdot A_{H,L/R} \tag{15a}$$

$$A^{\uparrow}_{R/L} \approx \frac{1 - R_{S,\uparrow}}{\left|1 - r_{S,\uparrow} r_H\right|^2} A_{H,R/L} \approx \left(1 - R_{S,\uparrow}\right) \cdot A_{H,R/L} \tag{15b}$$

where now *H* refers to coefficients consistent with a pure helices' layer inserted into a vacuum host, and *S* refers to the pure (lossless) substrate. The pedices *R/L* refer to left and right circular polarization, respectively, while the arrows indicating the input illumination direction. Lower *r* and upper *R* refer to complex reflection coefficient and reflectance, respectively. We have introduced the $|1-r_S{*}r_H|^2$ factor to include the effect of multiple reflections between the helices' layer and the substrate, although in the following we will neglect it for the sake of simplicity; indeed, this coefficient is extremely close to 1 and it would just give rise to negligible 2nd order effects. In addition to this, we will neglect scattered modes associated to wavelengths smaller than the horizontal *Λ* step of the helices' array. The helices' reflection spectra is non-dichroic, since dichroic effects appear only across transmitted fields [2].

The CD of absorption under downward illumination is:

$$CD^{\downarrow}_A = 2\frac{A^{\downarrow}_R - A^{\downarrow}_L}{A^{\downarrow}_R + A^{\downarrow}_L} \approx 2\frac{\left(A_{H,R} - A_{H,L}\right)\cdot\left(1 - R_{S,\downarrow} + R_H \cdot R_{S,u}\right)}{\left(A_{H,R} + A_{H,L}\right)\left(1 + \left(1 - R_H\right)\cdot R_{S,\downarrow}\right) - 2A_{H,R}\cdot R_{S,\downarrow}\cdot A_{H,L}} =$$
$$= CD_{A,H}\frac{1 - \left(1 - R_H\right)\cdot R_{S,\downarrow}}{1 + \left(1 - R_H\right)\cdot R_{S,\downarrow} - 2\cdot R_{S,\downarrow}\cdot\frac{A_{H,R}\cdot A_{H,L}}{A_{H,R} + A_{H,L}}} \tag{16}$$

while the one for upward illumination is:

$$CD^{\uparrow}_A \approx 2\frac{A^{\uparrow}_R - A^{\uparrow}_L}{A^{\uparrow}_R + A^{\uparrow}_L} = CD_{A,H} \tag{17}$$

Finally, the ratio between these CD expressions is:

$$\frac{CD^{\downarrow}_A}{CD^{\uparrow}_A} \approx \frac{1 - \left(1 - R_H\right)\cdot R_{S,\downarrow}}{1 + \left(1 - R_H\right)\cdot R_{S,\downarrow} - 2\cdot R_{S,\downarrow}\cdot\frac{A_{H,R}\cdot A_{H,L}}{A_{H,R} + A_{H,L}}} \tag{18}$$

In the following we show the reflection and dichroic absorption spectra of the helices layer, and the reflection spectra of the pure substrate under downward illumination. This latter is calculated neglecting multiple reflections internal to the Al_2O_3 layer, since usually they are not consistent with thick etalons like the Al_2O_3 bottom layer. Thus, the reflectance is calculated as:

$$R_{S,\downarrow} \approx R_{Multilayer} \tag{19}$$

The resulting spectrum of the CD_A ratio defined in (18) is plotted in figure S6.

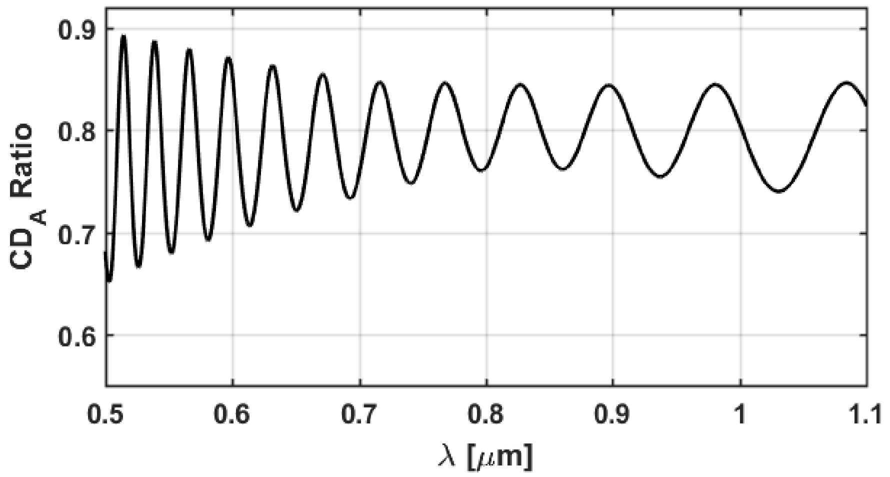

Figure S6. CD_A ratio spectrum. The average value is almost perfectly constant (0.79) along the entire observed spectrum, with oscillations due to the etalon effect of the GaN layer in the substrate.

To confirm our speculations, we performed also a second simulations' stage setting simultaneously 3 different cases of illuminations: downward illumination, upward illumination and the case of absent substrate (having the helix quite similar terminations, there is no need to make distinctions between illumination sides on this last case). Random variations to the helix geometrical parameters and an averaging procedure has been again applied as done in the main simulation stage.

Looking at figure S7, we see that the upward and vacuum CD_A profiles are quite similar each other, with upward CD_A appearing slightly stronger then downward CD_A, consistently with the prediction of the theory.

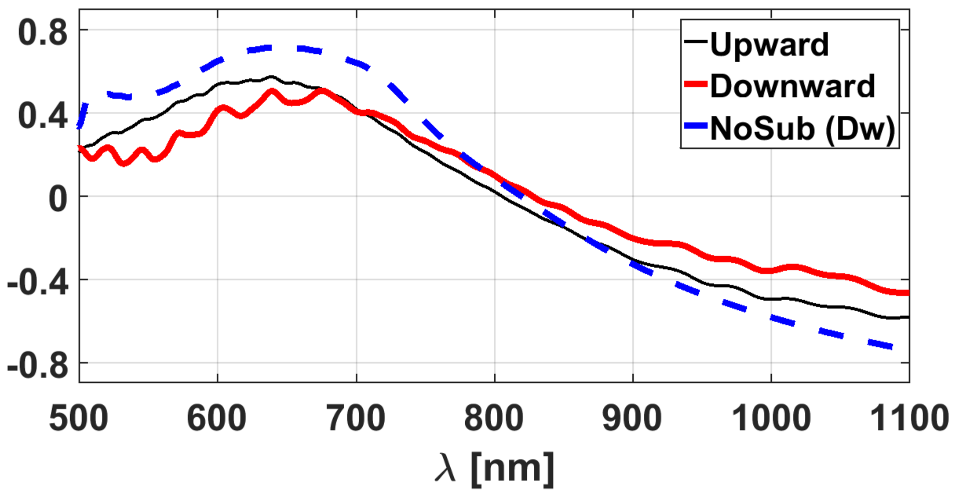

Figure S7. CD_A ratio spectrum for 3 different cases of illumination. The downward illumination case is evidently more affected by the etalon effects. Among the 2 cases where the substrate is involved, the upward illumination one is the closest to the no-substrate case in the range λ>~750nm (where scattered modes are perfectly absent).

The ratio between the 2 variants of CD_A is roughly 80% above the crossing region approximately located at λ~810nm. Below this wavelength (especially below 700nm, which is the array horizontal lattice period), diffraction orders start to affect the internal scattering process, and the overall response progressively diverges from the one of a homogeneous layer. See also the major spectral modulation of downward CD_A with respect to the upward and vacuum variant, consistently with the modulation of the CD_A-ratio function as defined in (18).

Other than this, we see that theoretically vacuum CD_A should be similar to the upward variant, but numerically the former appears to be slightly stronger than the latter. This can be explained by considering again that in our previous derivation we neglected superior diffraction orders (excited at wavelengths lower than the array lattice period); this difference is potentially enhanced by surface plasmon excitation internal to the helices layer; more precisely, this latter effect is weakened or strengthened according to the field localization and to the phase relations taking place inside the meta-structure [7].

Other differences are mainly due to the large set of random variations of geometrical parameters during the simulation stage which we performed to simulate the fabrication tolerances; the meaning of this step was to average the electromagnetic properties but added certain modifications on the final CD spectra.

References

1. Esposito, M. et al. Programmable Extreme Chirality in the Visible by Helix-Shaped Metamaterial Platform, *Nano Lett.* **16**, 9, 5823–5828 (2016)
2. Castanié, A., Mercier, J.-F., Félix, S., Maurel, A., Generalized method for retrieving effective parameters of anisotropic metamaterials, *Opt. Expr.* **22**, 24, 29937-29953 (2014)
3. Barker, A. S. Jr.; Ilegems, M. Infrared Lattice Vibrations and Free-Electron Dispersion in GaN, *Phys. Rev. B* **7**, 2, 743-750 (1973)

4. Pastrňák, J.; Roskovcová, L. "Refraction Index Measurements on AlN Single Crystals, *Phys. Stat. Sol.* **14**, 1, K5-K8 (1996)
5. Malitson, I. H.; Dodge, M. Refractive Index and Birefringence of Synthetic Sapphire, *J. Opt. Soc. Am.* **62**, 1405 (1972)
6. Esposito, M. et al. Triple-helical nanowires by tomographic rotatory growth for chiral photonics, *Nat. Comm.* **6**, 6484 (2015)
7. Tuccio, S.; Centini, M.; Benedetti, A.; Sibilia, C., *Jour. of Opt. Soc. Am. B* **30**, 2, pp.450-455 (2013)
